# Long Non-coding RNAs in Pathogenesis of Neurodegenerative Diseases

**DOI:** 10.3389/fcell.2021.719247

**Published:** 2021-08-30

**Authors:** Shiyue Zhou, Xiao Yu, Min Wang, Yujie Meng, Dandan Song, Hui Yang, Dewei Wang, Jianzhong Bi, Shunliang Xu

**Affiliations:** ^1^Department of Neurology, The Second Hospital, Cheeloo College of Medicine, Shandong University, Jinan, China; ^2^Department of Nutrition, The Second Hospital, Cheeloo College of Medicine, Shandong University, Jinan, China; ^3^Cheeloo College of Medicine, Shandong University, Jinan, China

**Keywords:** long non-coding RNAs, neurodegenerative diseases, Alzheimer’s disease, Parkinson’s disease, Huntington’s disease, amyotrophic lateral sclerosis

## Abstract

Emerging evidence addresses the link between the aberrant epigenetic regulation of gene expression and numerous diseases including neurological disorders, such as Alzheimer’s disease (AD), Parkinson’s disease (PD), amyotrophic lateral sclerosis (ALS), and Huntington’s disease (HD). LncRNAs, a class of ncRNAs, have length of 200 nt or more, some of which crucially regulate a variety of biological processes such as epigenetic-mediated chromatin remodeling, mRNA stability, X-chromosome inactivation and imprinting. Aberrant regulation of the lncRNAs contributes to pathogenesis of many diseases, such as the neurological disorders at the transcriptional and post-transcriptional levels. In this review, we highlight the latest research progress on the contributions of some lncRNAs to the pathogenesis of neurodegenerative diseases *via* varied mechanisms, such as autophagy regulation, Aβ deposition, neuroinflammation, Tau phosphorylation and α-synuclein aggregation. Meanwhile, we also address the potential challenges on the lncRNAs-mediated epigenetic study to further understand the molecular mechanism of the neurodegenerative diseases.

## Introduction

Epigenetics literally refers to regulation of gene expression due to external modifications to DNA and histones without altering DNA sequence. Caused by a variety of factors inside/outside of organisms, such as hormones, metabolism, diet, temperature, light, drugs, air pollution, age and so on, epigenetic changes could alter growth, development, reproduction and aging, and even contribute to pathogenesis of diseases such as neurological disorders. Significant efforts have been made in study of the epigenetic basis of neurodegenerative diseases (Thompson et al., 2020), such as Alzheimer’s disease (AD), Parkinson’s disease (PD), amyotrophic lateral sclerosis (ALS), Huntington’s disease (HD), and so on.

Epigenetic modifications could be classified into three main categories including DNA modifications, histone modifications, and non-coding RNAs (ncRNAs)-mediated modifications (Hwang et al., 2017; Thompson et al., 2020). Of the DNA methylation modifications, 5-methylcytosine (5-mC), 5-hydroxymethylcytosine (5-hmC), and *N*^6^-methyladenosine (m6A) have been identified and characterized as important epigenetic markers involved in regulation of gene expression in complicated mechanisms (Dermentzaki and Lotti, 2020; Pizzorusso and Tognini, 2020). In a broad sense, the ncRNAs are widely defined as all types of RNAs that are not translated into proteins due to the lack of an open reading frame (ORF).

Based on the length with 200 nt as cutoff value (Elling et al., 2016), ncRNAs could be classified into small ncRNAs (sncRNAs) and long ncRNAs (lncRNAs). Typical sncRNAs include microRNAs (miRNAs), piwi interacting RNAs (piRNAs), endogenous small interference RNAs (esiRNAs), micronucleus RNAs (snRNAs), small nucleolus RNAs (snoRNAs) and so on (Sosinska et al., 2015; Elling et al., 2016; Wu and Kuo, 2020).

**FIGURE 1:**
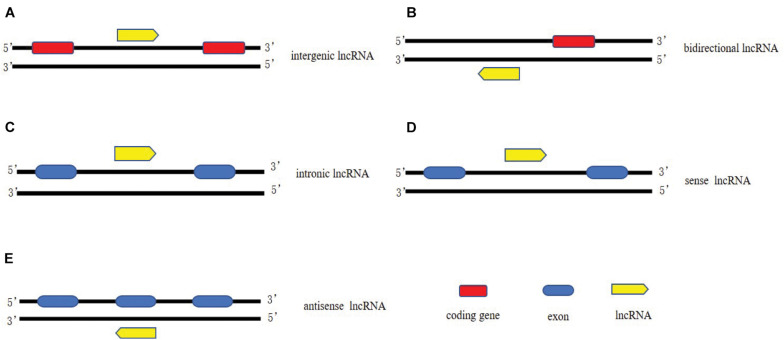
Classification of lncRNAs. **(A)** long intergenic non-coding RNAs, consisting of independent transcriptional units located between but not overlapped with protein codes; **(B)** bidirectional lncRNAs transcribed from different bidirectional promoters. **(C)** intron lncRNAs located in the intron region of protein coding genes (sense or antisense); **(D)** Sense lncRNAs are transcribed by the sense chain of the protein-coding gene, which overlaps with at least one exon of the protein-coding gene on the same chain and has the same transcriptional direction; **(E)** Antisense lncRNAs are transcribed by a complementary DNA chain of protein-encoded genes, they are transcribed in the opposite direction and overlaps with at least one exon of the positive gene.

It has been estimated that there are more than 50,000 of lncRNAs in the human genome (Managadze et al., 2013). Based on the genomic location of lncRNAs relative to protein coding genes, lncRNAs could be classified into five categories (Figure 1): (1) long intergenic non-coding RNAs (LincRNAs), consisting of independent transcriptional units located between but not overlapped with protein codes; (2) intron transcripts located in the intron region of protein coding genes (sense or antisense); (3) Sense lncRNAs are transcribed by the sense chain of the protein-coding gene, which overlaps with at least one exon of the protein-coding gene on the same chain and has the same transcriptional direction. The sense lncRNAs may partially overlap with the protein coding gene, or it may cover the entire sequence of the protein coding gene; (4) Antisense lncRNAs are transcribed by a complementary DNA chain of protein-encoded genes, they are transcribed in the opposite direction and overlaps with at least one exon of the positive gene; (5) bidirectional lncRNAs (bilncRNAs) transcribed from different bidirectional promoters (Quan et al., 2017; Liu et al., 2021).

Structurally, majority lncRNAs are very similar to but different from mRNAs. In terms of the similarity, they are transcribed from RNA polymerase II (POLII) from genomic sites in a chromatin-like state; usually 5-capped, spliced, and polyglandular. However, there is still a general trend to distinguish lncRNAs from mRNAs: lncRNAs tends to be shorter than mRNAs, with fewer but longer exons, failing to translate due to lacking in open reading frame, relatively low expression level and poor conservation of primary sequences (Quinn and Chang, 2015).

LncRNA could regulate expression of genes located in other genomic loci on different chromosomes at the transcriptional and posttranscriptional levels, such as RNA processing and translation (Elling et al., 2016). Most lncRNAs are located in the nucleus, thereby acting as scaffolds for chromatin modifiers by interacting with chromatin modification complexes or performing their regulatory functions as transcription co-regulators *via* binding to transcription factors (Ulitsky and Bartel, 2013). For example, LncRNA-XIST is a well-studied *cis-*acting LncRNA, which performs the critical developmental process of dosage compensation in females (Cerase et al., 2015). It suppresses the expression of the entire chromosome by inhibition the deposition of chromatin markers and relocating this inactive X chromosome to the periphery of the nucleus (Cerase et al., 2015). In the cytoplasm, lncRNAs usually act as regulators of RNA processing, such as RNA editing, splicing, and miRNA-mediated mRNA expression. LncRNAs also bind to other coding or ncRNAs to regulate transcription as competing endogenous RNAs (ceRNAs) (Salmena et al., 2011). LncRNAs can be used as a molecular sponge of miRNAs and titrate their levels, thereby increasing the expression of mRNAs targeted by these miRNAs (Salmena et al., 2011).

## Physiological Function of LncRNAs in the Central Nervous System

It is estimated that about 40% of LncRNAs are specifically expressed in brain tissue, and their number far exceeds that of protein-coding genes (Derrien et al., 2012). And compared with other tissues, LncRNAs are more conservative in brain tissue. In addition, in brain tissue, lncRNAs show stronger temporal and spatial specificity than mRNAs (Ponjavic et al., 2009). For example, LncRNAs have significant differences in different brain tissues (brain cortex, hippocampus, etc.) and in different age groups (Kadakkuzha et al., 2015). For example, human acceleration region 1 (HAR1), which is part of the overlapping lncRNA gene HAR1F (HAR1A). The gene is specifically expressed in Cajal–Retzius neurons in the developing human cerebral cortex (Pollard et al., 2006). LncRNAs are related to neural differentiation. In proliferative stem cells/progenitor cells, lncRNAs control the sequential activation of cell type-specific gene regulatory programs, thus promoting the development from pluripotent cells in early embryos to the terminal cell types evident in the brain of mature mammals (Zimmer-Bensch, 2019). LncRNA_DALI promotes neural differentiation by driving the expression of necessary neuronal differentiation genes in neuroblastoma cells through a variety of mechanisms. It promotes the expression of Pou3f3 in *cis*, forms a *trans-*acting regulatory complex with DALI, and regulates the expression of neural differentiation genes (Chalei et al., 2014). LncRNAs are related to neurite outgrowth, synaptogenesis and synaptic plasticity (Yang et al., 2021). Neurite outgrowth, synaptogenesis and synaptic plasticity all require complex gene expression regulation and signal transduction, in which lncRNA plays an important role (Modarresi et al., 2012; Mateos-Aparicio and Rodriguez-Moreno, 2019). Brain-derived neurotrophic factor (BDNF) is related to synaptogenesis and neurite outgrowth (Modarresi et al., 2012). BDNF-AS, the antisense LncRNA of BDNF, suppresses the BDNF growth factor gene by recruiting PRC2 to the BDNF site, thus affecting BDNF-mediated axonal growth, proliferation and apoptosis (Modarresi et al., 2012).

## LncRNAs and AD

Alzheimer’s disease is the most common, irreversible, and progressive neurodegenerative disorder, accounting for 60% of all dementia cases (Ryan et al., 2018), particularly the people over 65 years old. Among the more than 50 million cases of dementia patients worldwide in 2018, AD patients accounted for 50.75%. Statistically, the global cost of dementia reached United States $957.56 billion in 2015 and will continue increasing to United States $9.12 trillion in 2050 as estimated. The main pathological features of AD include β-amyloid plaque deposition, neurofibrillary tangles caused by hyperphosphorylated tau protein, and neuronal loss in specific areas of the brain (Lashley et al., 2018). Despite the high prevalence of AD, the etiology of AD remains to be unclear.

Emerging data have correlated the imbalance of lncRNA expression with a variety of human diseases, such as cardiovascular diseases, cerebrovascular diseases, malignant tumors and neurodegenerative diseases. Indeed, expression disorder of many lncRNAs has been detected in AD (Luo and Chen, 2016), mainly involved in A β deposition, Tau protein hyperphosphorylation, oxidative stress, neuroinflammation, mitochondrial dysfunction, autophagy regulation and other pathological processes (Table 1).

**TABLE 1:** Main dysregulated long non-coding RNAs in Alzheimer’s disease.

LncRNA	Target mRNA	AD-related process	Biological function	References
*BACE1-AS*	*BACE1*	Aβ deposition	Upregulating *BACE1* mRNA stability	Faghihi et al., 2008; Shah et al., 2012; Cortini et al., 2019
*XIST*	*BACE1*	Aβ deposition	Upregulating *BACE1* mRNA stability	Zhu et al., 2018; Yue et al., 2019
*51A*	*SORL1*	Aβ deposition	Downregulating *SORL1* variant A	Ciarlo et al., 2013
*Linc00507*	*MAPT/TTBK1*	Tau hyperphosp horylation	Regulating the expression of MAPT and TTBK1	Yan et al., 2020
*NEAT1*	*FZD3*	Tau hyperphosp horylation	Impairing the FZD3/GSK3β/p-tau signaling pathway	Zhao et al., 2020
*MALAT1*	*IL-10/SOC1*	Neuroin flammation	Regulating the expression of IL-10 and SOC1	Masoumi et al., 2019; Ma et al., 2019
*EBF3-AS*	*EBF3*	Neuronal apoptosis	Promoting neuronal apoptosis in AD	Magistri et al., 2015; Gu et al., 2018
*SNHG1*	*KRENEN1*	Neuronal apoptosis	Target miR-137 to inhibit KREMEN1	Causeret et al., 2016; Wang et al., 2019

### Aβ Plaque Formation

Previous studies have proven the contribution of lncRNAs to formation of the Aβ plaques derived from the proteolytic cleavage of Aβ precursor protein (APP) by β-position APP cleaving enzyme 1 (BACE1) and γ secretase. Indeed, dysregulation of BACE1 leads to overproduction of Aβ, contributing to pathogenesis of AD (Modarresi et al., 2011). Basically, lncRNA BACE1-AS is transcribed by RNA polymerase II from the antisense strand of the BACE1 locus located on chromosome 11. It was clear that BACE1-AS is highly expressed in brains of the AD patients and APP transgenic mice, regulates the expression of BACE1 mRNA and increases the production of Aβ (Cortini et al., 2019). The loss of BACE1 in animal models led to physical and behavioral defects, such as decreased learning ability, memory and emotional loss (Ma et al., 2007). The siRNA-based silencing of BACE1-AS in mouse brains significantly decreased the BACE1 mRNA levels in cortex, central and dorsal hippocampal regions (Faghihi et al., 2008). Mechanistically, high level expression of BACE1-AS could promote the stability of BACE1mRNA, which in turn enhances the processing ability of APP and consequently promotes Aβ deposition (Faghihi et al., 2008). Some epigenetic factors could regulate the expression of BACE1-AS. For example, stress response stimulates the expression of the BACE1-AS, accelerating development of AD (Shah et al., 2012).

Another important lncRNA essentially involved in the pathogenesis of AD is XIST, one of the most extensively characterized lncRNA, which plays important roles in cancer and cardiovascular diseases as well (Sun et al., 2017; Kong et al., 2018; Zhou et al., 2019). Expression of the XIST was upregulated in AD model mice and N2a cells in response to H_2_O_2_ oxidative stress. Bioinformatic analysis predicted a binding site between miR-124 and XIST as well as between miR-124 and BACE1. Expression of XIST was negatively correlated with miR-124 but positively with BACE1. Consistently, knockdown of XIST upregulated expression of miR-124 but downregulated the expression of BACE1 in N2a cells. The direct interactions between XIST and miR-124, as well as BACE1 and miR-124 were further confirmed by using luciferase reporter assay (Zhu et al., 2018). Altogether, it could be concluded that silencing of XIST could attenuate alteration of the AD-related BACE1 *via* the miR-124/BACE1 signaling pathway (Yue et al., 2019). Besides BACE1-AS and XIST, lnc-51A has been acknowledged as an important factor for Aβ deposition. Upregulation of the lnc-51A was detected in plasma of sporadic AD patients. Furthermore, expression of lnc-51A was negatively correlated with disease progression assessed by MMSE score most probably because the upregulation of lnc-51A altered splicing mode of SORL1, thereby causing damage to APP processing and leading to promotion of the Aβ deposition (Ciarlo et al., 2013). These results suggest that the lnc-51A may serve as a stable diagnostic biomarker of AD as well.

### Tau Hyperphosphorylation

As another important possible pathogenesis of AD, Tau plays an important role in inducing Aβ deposition. Hyperphosphorylation of Tau leads to tau aggregation, exerting neurotoxicity that destroys neuronal function and thereby inducing AD (Clavaguera et al., 2009; Zempel and Mandelkow, 2015). So far, several lncRNAs have been identified to be involved in Tau hyperphosphorylation, such as linc-00507, lncRNA SOX21-AS1, and NEAT1. Expression of linc-00507 was significantly upregulated in hippocampus, cerebral cortex, and AD SH-SY5Y-like cells of APP/PS transgenic mice. The linc-00507 could regulate the expression of microtubule-associated proteins Tau (MAPT) and tau-tubulin protein kinase 1 (TTBK1) *via* two mechanisms: as competitive endogenous RNA (ceRNA) to bind miR181c-5p, as enhancer of hyperphosphorylation of tau protein by activating P25/P35/GSK3β signaling pathway (Yan et al., 2020).

Upregulation of SOX21-AS1 was also observed in SH-SY5Y and SK-N-SH cells treated with Aβ 1–42. Essentially, the SOX21-AS1 functions as a sponge for miR-107 in SH-SY5Y and SK-N-SH cells. Silencing of SOX21-AS1 could attenuate the level of Tau phosphorylation mediated by Aβ 1–42 in SHSY5Y and SK-N-SH cells. Consistently, the SOX21-AS1 knockdown reversed the level of p-Tau mediated by miR107 inhibition (Xu et al., 2020a). More importantly, SOX21-AS1 silencing could inhibit hippocampal neuronal apoptosis and enhance memory and learning ability in AD mice (Zhang et al., 2019a), suggesting that SOX21-AS1 could serve as a potential biomarker for AD patient.

Similarly, NEAT1 was upregulated in SH-SY5Y cells and primary neurons of the AD model, and downregulation of NEAT1 promoted Tau protein phosphorylation through FZD3/GSK3 β/p-tau pathway (Zhao et al., 2020).

### Neuroinflammation

The neuroinflammation has been acknowledged as one of the key features in AD with a primary role in exacerbation of Aβ plaques and tau hyperphosphorylation, contributing to AD pathology. So far, some lncRNAs have been identified to be involved in the neuroinflammation, such as MALAT1.

Downregulation of MALAT1 promotes the polarization of macrophages to M1 phenotype and induces the proliferation of T cells in multiple sclerosis (MS), indicating the potential anti-inflammatory effect of MALAT1 on MS (Masoumi et al., 2019). Stimulation of SD rat embryonic PC12 cells and primary cortical neurons with nerve growth factor (NGF) inhibited apoptosis of neurons in PC12 AD model and primary neuronal AD model mediated by lncMALAT1 and promoted growth of neurites in primary neuronal AD model. To further explain the mechanism of MALAT1 on neuroinflammation in AD, it was found that MALAT1 downregulated expression of IL-6 and TNF-α in PC12AD model and primary neuronal AD model, but enhanced expression of IL-10. These results suggest that MALAT1 contributes to the pathogenesis of AD by regulating inflammation response. Further study demonstrated that MALAT1 may inhibit several inflammation-related miRNAs, such as miR-125b and miR-155. These miRNAs targets to the genes involved in many signaling pathways, such as JAK signal transduction and activator of transcription (STAT) pathway, nuclear factor- kB (NF-kB) signaling pathway, JNK pathway and p38 signaling pathway, thereby inhibiting neuroinflammation of AD (Ma et al., 2019).

LncRNA MEG3 (maternally expressed gene 3) plays important roles in cell proliferation, learning, and memory (Guo et al., 2016; Zhang et al., 2016a). The expression of MEG3 was downregulated in the hippocampus of rats with AD. It has been found that MEG3 upregulation could improve the spatial learning and memory ability of AD rats. MEG3 can reduce the deposition of Aβ 25–35 and oxidative stress in the hippocampus of rats with AD, and reduce the inflammatory injury by downregulating IL-1 β, IL-6 and TNF- α. In addition, the overexpression of MEG3 inhibits the activation of astrocytes in the hippocampus of patients with AD by inhibiting PI3K/Akt signal pathway (Yi et al., 2019).

### Neuronal Apoptosis

LncRNA-mediated neuronal apoptosis has been linked to pathogenesis of AD. Knockout of SNHG1 could increase cell viability and inhibit apoptosis in SH-SY5Y and HPN cells treated with Aβ 25–35. The SNHG1 could protect SH-SY5Y and HPN cells treated with Aβ 25–35 from neuronal apoptosis *via* inhibiting miR-137. KREMEN1 belongs to the dickkopf (Dkk) family of Wnt antagonists. Wnt-independent way plays a role in promoting apoptosis in cells (Causeret et al., 2016). Further study showed that knockdown of SNHG1 exerts its neuronal protective effect through the inhibition of KRENEN1 by ceRNA miR-137 (Wang et al., 2019).

**FIGURE 2:**
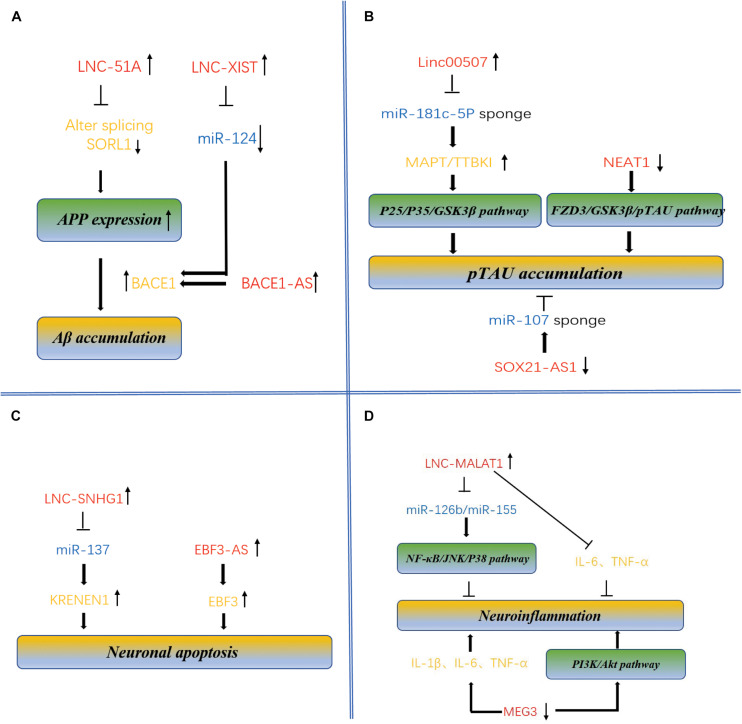
Molecular mechanism of lncRNAs and miRNAs mediated accumulation of Aβ plaques and pTAU, neuronal apoptosis and neuroinflammation in AD. **(A)** Dysregulation of BACE1-AS, XIST, and lnc-51A leads to overproduction of Aβ, contributing to pathogenesis of AD. BACE1-AS, XIST, and lnc-51A are highly expressed in brains of the AD patients and APP transgenic mice, regulates the expression of BACE1 mRNA and increases the production of Aβ. Enhanced expression of lnc-51A in AD patient altered splicing mode of SORL1, thereby causing damage to APP processing and leading to promotion of the A β deposition. XIST and BACE1 are targets of miR-124 because there is a binding site in these two targets. The XIST-miR-124-BACE1 axis regulates the expression of BACE1 in AD, determining the formation of Aβ. **(B)** LncRNAs regulate pTAU accumulation. Linc-00507, SOX21-AS1, and NEAT1 have been characterized to be involved in Tau hyperphosphorylation. Significantly upregulated expression of linc-00507 downregulates miR181c-5p, thereby elevating the levels of MAPT and TTBK1 and consequently enhancing hyperphosphorylation of tau protein by activating P25/P35/GSK3β signaling pathway. On the other hand, silencing of SOX21-AS1 could attenuate the level of Tau phosphorylation mediated by miR107 inhibition. Similarly, downregulation of NEAT1 promoted Tau protein phosphorylation *via* FZD3/GSK3 β/p-tau pathway. **(C)** LncRNAs regulate neuronal apoptosis. LncRNA-SNHG1, lnc-EBF3-AS, and MEG3 have been identified to be involved in neuronal apoptosis, linking to pathogenesis of AD. SNHG1 silencing inhibited miR-137, therefore repressing KRENEN1 expression and increasing cell viability and inhibiting apoptosis. was maladjusted in late-onset AD patients (Magistri et al., 2015), its upregulation of lnc-EBF3-AS stimulated the expression of EBF3, thus promoting the apoptosis of neurons in AD. **(D)** LncRNAs regulate neuroinflammation. Some lncRNAs, and miRNAs such as MALAT1 and MEG3, have been identified to be involved in the neuroinflammation. Upregulation of MALAT1 downregulated expression several inflammation-related miRNAs, such as miR-125b and miR-155. These miRNAs target to the genes involved in many signaling pathways, such as JAK signal transduction and activator of transcription (STAT) pathway, nuclear factor –kB (NF-kB) signaling pathway, JNK pathway and p38 signaling pathway, thereby inhibiting neuroinflammation of AD. downregulated expression of MEG3 promoting the occurrence of inflammation.

Lnc-EBF3-AS was maladjusted in late-onset AD patients (Magistri et al., 2015), its upregulation was detected to stimulate the expression of EBF3 in the hippocampus of the AD transgenic mouse model, thus promoting the apoptosis of neurons in AD (Gu et al., 2018). This study suggests the potential role of the lnc-EBF3-AS in the pathogenesis of AD (Figure 2).

## LncRNAs and PD

Parkinson’s disease is an anther age-related progressive neurodegenerative disease, affecting 1% of people mainly over the age of 65. Compared with effect on memory, thinking and cognition in AD, PD mainly affects the motor system. The two typical features of PD include loss of dopaminergic neurons in the substantia nigra and accumulation of a-synuclein (Calabresi et al., 2013; Bose and Beal, 2016). Despite decades of research, our understanding to the pathophysiology and diagnosis of PD is still at infancy stage. Accordingly, so far, there is no efficient strategy for therapy of the disease albeit the current dopamine replacement strategies and surgical interventions can provide symptom relief, still failing to prevent or reverse the underlying pathology (Hegarty et al., 2020; Table 2).

**TABLE 2:** Main dysregulated long non-coding RNAs in Parkinson’s disease.

LncRNA	Target mRNA	AD-related process	Biological function	References
*H19*	*HPRT1*	Dopaminergic neuron loss	Upregulating *HPRT1* mRNA stability	Li et al., 2016; Jiang et al., 2020
*NEAT1*	*LRRK2*	Dopaminergic neuron loss	Regulation of oxidative stress in dopaminergic neurons	Bastias-Candia et al., 2019; Simchovitz et al., 2019
*SNHG1*	*SIAH1*	α-synuclein aggregation	Promoting the ubiquitination of α-synuclein	Lee et al., 2008; Chen et al., 2018
*LincRNA-p21*	*α-Synuclein*	α-synuclein aggregation	Transcription repressor	Xu et al., 2018
*GAS5*	*NLPR3*	Neuroin flammation	Promoting inflammation of microglia	Xu et al., 2020b
*HAGLROS*	*PI3K*	Autophagy	Regulating the PI3K/AKT/mTOR signaling pathway	Peng et al., 2019

### Dopaminergic Neuron Loss

Efforts have been made to elucidate the mechanisms of the lncRNA-mediated dopaminergic neuron loss in AD (Jiang et al., 2020). H19, among the first lncRNAs identified, has been considered as one of the major players in embryonic development, cancer, and PD *via* regulating proliferation, differentiation and cell motility through controlling DNA methylation and intracellular miRNA pattern being both a sponge for miRNAs and miRNA reservoir.

H19 is located in the imprinted gene cluster H19-IGF2 (Li et al., 2016), whereas the IGF2- proinsulin precursor (INS)-TH gene cluster located at the telomere end of chromosome 11 has been reported to encode various proteins important for homeostasis in dopamine neurons (Sutherland et al., 2008). Therefore, this gene cluster is associated with the risk of PD (Sutherland et al., 2008). Level of the H19 significantly decreased in the 6-hydroxydopa (6-OHDA)-induced PD mice. Consistently, overexpression of the H19 could prevent dopaminergic neuron loss in the 6-OHDA-induced PD model by activating HPRT1-mediated Wnt/β catenin signaling pathway *via* impairing miR-301b-3p-targeted repression of HPRT1 transcription (Jiang et al., 2020). Indeed, significant upregulation of miR-301b in the pars compacta of substantia nigra of PD mouse model downregulates HPRT1 expression. Thus, by serving as sponge of miR-301b-3p, H19 could reduce the loss of dopaminergic neurons and eventually slow down the degeneration of striatum nigra (SN) (Jiang et al., 2020).

NEAT1 upregulation in dopaminergic neurons of PD may protect dopaminergic neurons from LRRK2-mediated damage under the oxidative stress. Highly variable expression level of NEAT1 in SN may reflect its response to multiple factors. Due to the estrogen-associated high expression of NEAT1 in female, dopaminergic neurons in the SN could be protected, potentially explaining why the incidence of PD in women is significantly lower than in men (Chakravarty et al., 2014; Bastias-Candia et al., 2019). However, significant upregulation of NEAT1 expression does not always protect dopaminergic neurons for any PD patients. Instead, it is in a patient (such as gender and carriers of LRRK2 mutations) dependent manner. For example, the enhanced NEAT1 expression in simvastatin and fenofibrate was harmful for certain PD patients, such as carriers of LRRK2 mutations. Altogether suggest that certain drugs that could alter the expression of NEAT1 in SN might potentially function as prevention or therapy of PD for a specific subpopulations of PD patients (Simchovitz et al., 2019).

Another important lncRNA that could be potentially involved in Dopaminergic neuron loss is SNHG14, its expression is upregulated in brain tissue of PD mouse model induced by rotenone. Binding affinity of the transcription factor SP-1 to SNHG14 promoter was enhanced, leading to upregulation of SNHG14 expression in the rotenone induced PD model of MN9D cells. Consistently, knockdown of SNHG14 expression in MN9D cells could alleviate the damage induced by rotenone in dopaminergic neurons through activation of the miR-133b inhibited by SNHG14, whereas miR-133b targets to 3′UTR of α-synuclein that contributes to PD pathogenesis. Similarly, the silencing of SNHG14 reduced the neuronal damage in the PD mouse model as well. Therefore, silencing of SNHG14 reduces the damage of dopaminergic neurons by downregulating α-synuclein *via* miR-133b, improving the symptoms of PD (Zhang et al., 2019b).

### α-Synuclein Aggregation

The α-synuclein, a presynaptic neuron protein, is a major component of Lewy bodies and has been linked to several neurodegenerative diseases (Bennett, 2005). Mutations in the α-synuclein gene are associated with the pathophysiology of PD. The abnormally soluble oligomeric conformation of α-synuclein is considered to be a toxic, leading to neuronal death and disruption of cell homeostasis. Targeting α-synuclein is generally considered as a potential strategy for PD therapy (Dehay et al., 2015).

As mentioned above, MALAT1 confers neuroinflammation in AD. However, in PD, instead of leading to neuroinflammation, MALAT1 was related to the aggregation of α-synuclein. The expression of MALAT1 is upregulated in the midbrain of MALAT1 -induced PD mouse model and in SH-SY5Y cells exposed to MPP+, suggesting that MALAT1 may play an important role in the pathogenesis of PD. MALAT1 binds to α-synuclein to enhance its stability, resulting in high abundance of α-synuclein. It is well known that β-asarone plays a neuroprotective role by mediating the downregulation of α-synuclein and MALAT1. In a *in vivo* PD model, β-asarone could increase the number of TH+ cells and downregulate expression of α-synuclein, while the overexpression of MALAT1 could reverse its effect (Zhang et al., 2016b). To understand the pathophysiology of PD models induced by MPP+ and MPTP, miRNA profiling was conducted and found that miR-15b-5p expression was downregulated in response to MPP+ and MPTP accompanied by upregulation of SNHG1 expression.

On another hand, it has been reported that SIAH1 interacts with the brain-rich E2 ubiquitin-binding enzyme UbcH8 to promote the ubiquitination of α-synuclein, thereby promoting the aggregation and toxicity of α-synuclein (Lee et al., 2008) in neurons. Interestingly, SIAH1 serves as one of the downstream targets of miR-15b5p, and upregulation of miR-15b-5p repressed the accumulation of α-synuclein and inhibited apoptosis induced by α-synuclein in SH-SY5Y cells. In contrast, overexpression of SIAH1 or downregulation of miR-15b-5p reversed the protective effect of siSNHG1 on SH-SY5Y cells due to α-synuclein aggregation. Therefore, it is further concluded that SNHG1 promotes the aggregation and toxicity of α-synuclein through activating SIAH1 in SH-SY5Y cells *via* downregulating miR-15b-5p (Chen et al., 2018). In addition to SNHG1, a long intergenic non-coding RNA-p21 (lincRNA-p21) with a length of 3,100 nt located on chromosome 6, also play a part in cell proliferation, metabolism and reprogramming, and lincRNA-p21 is considered as a potential diagnostic marker for many diseases (Chen et al., 2017). The lincRNA-p21 is a p53-dependent transcriptional target gene that functions as a transcriptional inhibitor and triggers apoptosis (Huarte et al., 2010). In the MPTP-induced PD mice and the MPP+-treated SH-SY5Y cells, lincRNA-p21 expression was significantly upregulated, in accordance with inhibition of cell viability and induction of apoptosis associated with downregulation of miR-1277-5p and upregulation of α-synuclein protein (Xu et al., 2018). Altogether addresses the regulation of α-synuclein aggregation and toxicity by axis of SNHG1-miR15b-5p- SIAH1-linc-p21- miR-1277-5p- α-synuclein aggregation.

### Neuroinflammation

Neuroinflammation is considered to promote the development of PD (Kaur et al., 2017). As the main immune cells of CNS, microglia are activated in PD models in response to MPTP or lipopolysaccharide (LPS) (More and Choi, 2017), mediating the immune response in the brain and triggering the release of pro-inflammatory cytokines such as tumor necrosis factor-α (TNFα), IL-1β and IL-6, and consequently leading to apoptosis and death of DA neurons in the midbrain (Tang and Le, 2016).

Some lncRNAs are involved in activation of microglia such as SNHG1 and lnc-GAS5. Upregulation of SNHG1 has been observed in both LPS-stimulated BV2 microglia and MPTP-induced PD mice, suggesting that this lncRNA is related to LPS-induced activation and inflammation of BV2 microglia. In the MCS metastasis model, downregulation of SNHG1 could prevent the apoptosis of activated microglia and reduce the activation of microglia and the loss of dopaminergic neurons in PD mice induced by MPTP. Moreover, SNHG1 as the ceRNA of miR-7, regulates the expression of NLRP3, leading to the activation of NLRP3 inflammatory bodies (Cao et al., 2018). More data showed that by spongifying miR223-3p, the GAS5 could also promote microglial inflammation in PD *via* regulation of NLRP3 pathway (Xu et al., 2020b).

### Autophagy

Increasingly emerged evidence supports the view that autophagy dysfunction contributes to occurrence or development of PD (Ghavami et al., 2014). For example, overexpression of transcription factor EB (TFEB), a key autophagy regulator, helps to clean the α-synuclein aggregation and to prevent neuronal loss in PD (Decressac et al., 2013).

After death autopsy showed significant upregulation of the SNHG1 in PD patient brain samples (Kraus et al., 2017). Consistent with PD patients, substantial increase of SNHG1 expression *in vivo* and *in vitro* were detected the MPP+-treated MN9D cells. Bioinformatic analysis confirmed a common miR-221/222 response element specifically for SNHG1. Interestingly, overexpression of miR-221/222 promoted the level of LC3-II and reduced the neurotoxicity induced by MPP+, in consistence with the increased the level of LC3-II in MN9D cells under SNHG1 silencing, thereby preventing cytotoxicity and suggesting the regulation of SNHG1 through miR-221/222. In MPP+-treated MN9D cells, miR221 or miR-222 inhibitors reversed siRNA-SNHG1-induced p-mTOR inhibition, which was consistent with the effect of silencing p27. This experiment finally confirmed that lncRNA-SNHG1 regulates the expression of p27/mTOR through competitive interaction with miR-221/222 members in MPP+-treated MN9D cells (Qian et al., 2019).

Another lncRNA HAGLROS is considered to be involved in occurrence and development of PD. Upregulated expression of HAGLROS in PD mouse model induced by MPTP and SH-SY5Y cells poisoned by MPP, is in consistence with the reduction of apoptosis and autophagy in response to silencing of lnc-HAGLROS. Further *in vitro* studies showed that HAGLROS negatively regulated the expression of miR-100, and that HAGLROS enhanced apoptosis and autophagy of SH-SY5Y cells exposed to MPP through downregulation of sponge-like miR-100. These findings indicated that the inhibition of HAGLROS could alleviate the MPP induced damage of SH-SY5Y cells by activating PI3K/AKT/mTOR pathway, suggesting the essential role of lnc-HAGLROS in PD (Peng et al., 2019).

**FIGURE 3:**
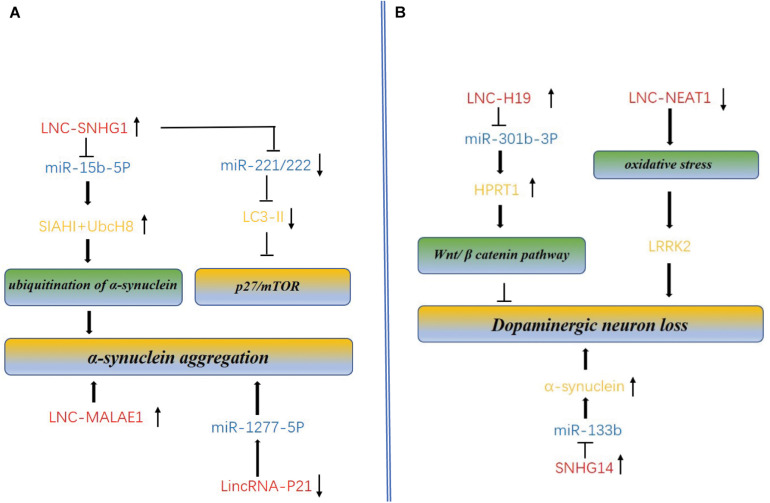
Molecular mechanism of lncRNAs and miRNAs mediated accumulation of dopaminergic neuron loss, α-synuclein aggregation and autophagy in PD. **(A)** LncRNAs regulate α-synuclein aggregation and autophagy. SNHG1 and lnc-MALAE1 are highly expressed in AD, promotes the α-synuclein aggregation. SNHG1 also can inhibit autophagy by reducing the expression of mTOR. Linc-P21 is down-expressed in PD and promotes aggregation through overexpression of miR-1277-5P. **(B)** LncRNAs regulate dopaminergic neuron loss. Overexpression of the H19 could prevent dopaminergic neuron loss in the 6-OHDA-induced PD model by activating HPRT1-mediated Wnt/β catenin signaling pathway *via* impairing miR-301b-3p-targeted repression of HPRT1 transcription. NEAT1 upregulation in dopaminergic neurons of PD may protect dopaminergic neurons from LRRK2-mediated damage under the oxidative stress. Overexpression of the ln_SNHG14 could promote dopaminergic neuron loss.

Instead of promoting MPP+-induced apoptosis as did the HAGLROS, upregulation of NORAD could protect the apoptosis and mitochondrial dysfunction, while the downregulation of NORAD could reverse the protection (Tatton et al., 2003). To further understand the protection mechanism of the NORAD, *in vitro* cytotoxicity assays were conducted by generating stable SHSY5Y cell lines that silence or overexpress NORAD mediated by lentivirus in the SHSY5Y cells. Overexpression of NORAD could significantly protect SH-SY5Y cells from the MPP+-induced cytotoxicity, while the down-regulation enhanced the cytotoxicity in a MPP+ dose and time dependent manner (Song et al., 2019) (Figure 3).

## LncRNAs and ALS

As a common and serious adult-onset neuromuscular disease, ALS affects motor neurons in the spinal cord, brainstem and motor cortex. Sporadic ALS(SALS) accounts for up to 90% of ALS cases and the other 10% have a strong genetic component also called as familial ALS (FALS). Mutations in more than 20 genes contribute to FALS (Renton et al., 2014). TDP-43, a multifunctional RNA binding protein encoded by TARDBP gene, is considered to be the main component involved in ALS. TDP-43 mutations contribute to about 95% of SALS cases, while FALS cases are caused by C9ORF72 gene mutations. However, the pathological boundary is not that clear between SALS and FALS. For example, dozens of TARDBP mutations have been found in both FALS and SALS patients. Further characterization of all these TDP-43 mutation cases *via* loss of TDP-43 from the nucleus and cytoplasmic and gain of TDP-43 function suggests that this protein is related to ALS (Shelkovnikova et al., 2018; Table 3).

**TABLE 3:** Main dysregulated long non-coding RNAs in Huntington’s disease and amyotrophic lateral sclerosis.

Disease	LncRNA	Target mRNA	Biological function	References
Amyotrophic lateral sclerosis	*NEAT1/MALAT1*	TDP-43	Improving toxicity of TDP-43	Hutchinson et al., 2007; Tollervey et al., 2011; Militello et al., 2018
	*MEG3*	Hox	promotes the formation of PRC2-Jarid2 complexes	Yen et al., 2018; Vangoor et al., 2020
	*C9ORF72-AS*	C9ORF72	Regulates repetition of (G4C2)n in C9ORF72	Kovanda et al., 2015; DeJesus-Hernandez et al., 2017; Shelkovnikova et al., 2018
Huntington’s disease	*NEAT1*	HTT/TP53	Reducing the expression of endogenous HTT and TP53.	Chanda et al., 2018
	*TUG1*	P53	Regulates P53 transcriptional regulatory pathway in HD	Khalil et al., 2009

### LncRNAs Interactive With TDP-43

Transcriptomic profiling conducted in 30 patients with SALS and 30 matched normal persons, led to identification of 293 of lncRNAs differentially expressed (DE) in the SALS patients. Of the DE lnc-RNAs, 183 out of 293 (62.5%) were upregulated, while 184 out of 293 (62.8%) belong to antisense RNAs and 81 out of 293 (27.7%) were reported as real DE lincRNAs (Gagliardi et al., 2018), suggesting the lncRNAs mediated regulation of ALS.

NEAT1 containing a sequence rich in GpCs, mainly expressed in spinal motoneurons at the early stage of ALS and contributed to pathogenesis of ALS. The NEAT1 mainly binds to TDP43 in the brain tissue of ALS patients and cultured cells (HeLa and SH-SY5Y) (Tollervey et al., 2011). Both TDP-43 and fused in sarcoma/translocated in liposarcoma (FUS/TLS) are considered to be necessary components for the formation of normal accessory spots *via* direct protein-protein interactions (Hutchinson et al., 2007). The frequency of accessory spot formation increased dramatically at the early stage of ALS. Therefore, NEAT1 was considered to be the scaffold of RNA binding proteins in the motor neuron nuclei of ALS patients. As a component of Paraspeckles, FUS significantly promotes their stability by regulating the steady-state level of NEAT1 and maintaining the structure of the nucleosome (Naganuma et al., 2012). More detailed analysis showed that NEAT1 was highly enriched in neurons of the anterior horn of the spinal cord and in the cortex of ALS patients (Tollervey et al., 2011; Shelkovnikova et al., 2018). Given that the formation of accessory spots in the spinal cord of SALS and FALS patients is much more than that in the healthy people, it is plausible to speculate that the accessory spot formation might be a common feature of ALS patient (Shelkovnikova et al., 2018).

Other lncRNAs are involved in ALS mostly *via* interaction with proteins related to ALS pathogenesis, such as TDP-43 or FUS. The expression of the MALAT1 was significantly upregulated in the cortex of sporadic FTD patients and binds to TDP-43 that interacts with several other lncRNAs, including lnc-BDNFOS and lnc-TFEB α (in SHSY5Y cells) and lnc-Myolinc (in muscle cells) (Militello et al., 2018). The expression of heat shock rna ω (hsr ω) in lncRNA is positively regulated by TDP-43 *via* direct interaction with hsr ω. Upregulated expression of hsr ω is detected in both human FTD patients and cellular model of TDP-43 overexpression (Chung et al., 2018). The ALS-related mutations in FUS affect expression of several lncRNAs in mESC- MN. For example, relative to wild type FUS-/-MNS, mutation FUSP517L/517L could upregulate lnc-Lhx1os specifically, while both lnc-MN-1 (2610316D01Rik) and lnc-MN2 (5330434G04Rik) were downregulated, indicating that the loss of FUS function altered the expression of several lncRNAs.

Evolutionally, lnc-Lhx1os, lnc-MN-1 and lnc-MN2 are conserved between mice and humans, and their expressions levels are elevated during the differentiation of human MN *in vitro* (Biscarini et al., 2018). The homologous gene dFU in Drosophila and human FUS both interact with hsr ω, and its deletion could lead to cytoplasmic dislocation and loss of nuclear dFU function. In addition, knockout of MN-specific hsr ω impaired the movement of larvae and adults of Drosophila melanogaster, and also led to the anatomical defects of MN presynaptic terminals. In view of the different interactions between TDP-43/FUS and lncRNAs, the cytoplasmic mislocalization and dysfunction of TDP-43 and FUS in ALS may affect the distribution, expression and/or function of lncRNAs, thereby leading to degeneration and ALS of MN (Lo Piccolo and Yamaguchi, 2017).

### LncRNAs Derived From C9ORF72 Antisense Transcript

Expansion of (GGGGCC)n (G4C2) repeats in 5′UTR of the C9ORF72 gene has been acknowledged as one of the most common genetic factors in ALS (Shelkovnikova et al., 2018). Three transcript variants were detected in the expansion of the (G4C2)n repeats in C9ORF72, including variants 1 and 3 located in intron 1, and variant 2 in the promoter region (Balendra and Isaacs, 2018). The (G4C2)n repeats in C9ORF72 were observed in nearly 40% of FALS and FTD cases and in up to 8% of SALS, respectively (Majounie et al., 2012; DeJesus-Hernandez et al., 2017). As for the (G4C2)n repeat number, healthy persons carry as many as 20–30 repeats, while the repeat number was expanded to hundreds even thousands in ALS (DeJesus-Hernandez et al., 2017). The C9ORF72-associated ALS cases (C9-ALS) are associated with loss of C9ORF72 function and acquisition of toxic function mediated by expansion of (G4C2)n repeats. C9ORF72 antisense transcript (C9ORF72-AS) is a kind of lncRNA named as lnc-C9ORF72-AS transcribed from intron 1 of the C9ORF72 gene (Mori et al., 2013). Although C9ORF72 and its corresponding proteins have been extensively investigated, the functional correlation of lnc-C9ORF72-AS remains poorly understood. C9ORF72-S can form a G-quadruple known to regulate gene expression (Reddy et al., 2013; Haeusler et al., 2014). Theoretically, C-rich C9ORF72-AS repeats may not form a similar structure. On the contrary, the expansion of (G2C4)n repeats in lnc-C9ORF72-AS may form a C-rich sequence (Kovanda et al., 2015), thereby potentially affecting the stability and transcription of the genome. Furthermore, it was found that these could rescue disease-specific transcriptional changes in iPSC-derived neurons by using antisense oligodeoxynucleotides (ASO) against C9ORF72 in Drosophila model (Sareen et al., 2013; Zhang et al., 2015).

## LncRNAs and Huntington’s Disease

Huntington’s disease is a neurodegenerative disease characterized by autosomal dominant inheritance, cognitive impairment, dance motor disorder and mental disorder. Selective loss of intermediate spinous neurons in caudate nucleus and putamen is a typical pathological feature of HD (Labbadia and Morimoto, 2013). The abnormal expansion of (CAG)n in HT protein gene leads to the formation of mutant HT proteins containing the expanded polyglutamine region. The mutant HT protein induces neurodegeneration through a variety of mechanisms such as transcriptional disorders, clearance of misfolded proteins, toxic N-terminal fragments, mitochondrial dysfunction and oxidative stress (Ross and Tabrizi, 2011; Sunwoo et al., 2017). Given that HT protein is widely expressed in different cell types at all developmental stages of the body, the pathogenesis of HD may begin early and last for a lifelong process (Bassi et al., 2017; Table 3).

Previous studies have confirmed the contribution of ncRNAs to HD pathogenesis. 12 of ncRNAs were identified and characterized in the brains of HD mice, eight of which are human homologs. Of the identified ncRNAs, MEG3, NEAT1 and XIST showed a sustained and significant increase in HD cells and animal models. Knockdown of MEG3 and NEAT1 in HD cell model resulted in a significant decrease in the aggregation of mutant HT protein and a downregulation of endogenous Tp53 expression (Chanda et al., 2018). Upregulation of MEG3 was observed at the early stage (6 weeks) and lasted to late stage (8 weeks) in R6/2 mice as well as in other HD cell models. However, downregulation of MEG3 was also reported in the brains of HD patients (Johnson, 2012).

Previous studies reported elevation of the NEAT1 levels in both GEO data mining, HD models, and autopsy samples of HD patients (Brochier et al., 2008; Makhlouf et al., 2014). However, no information is available for the alteration of XIST levels in HD (Chanda et al., 2018). Other studies have shown that NEAT1 physically interact with loci of other genes located on the active chromatin sites near the Tp53 gene (Yang et al., 2013). Tp53 directly regulates the transcription of NEAT1 by binding to the promoter region of the gene (West et al., 2014; Sunwoo et al., 2017). Therefore, the level of NEAT1 and Tp53 is controlled by the feedback loop. Given that inhibition of proteasome degradation could increase levels of NEAT1, cytoplasmic and nuclear polymers containing ubiquitin proteins are expected to be identified as well (Idogawa et al., 2017). In view of the fact that proteasome degradation is affected in HD (Hirose et al., 2014; Hyrskyluoto et al., 2014) and that NEAT1 level is elevated in HD (Makhlouf et al., 2014), it could be inferred that Neat1 may affect proteasome degradation in HD. Aberrant expression of lncRNAs was observed in the postmortem brain of human HD and the brain of R6Unix2 mice, such as upregulation of NEAT1. Transfection of the short isomer of NEAT1 into N2a cells significantly reduced cell death caused by H_2_O_2_ oxidative stress, revealing the functional correlation of NEAT1 and neuroprotective mechanism in the pathogenesis of HD (Sunwoo et al., 2017).

MEG3, NEAT1, and XIST may interact with many miRNAs through sequence complementarity, potentially reducing the efficiency of binding to their target mRNAs by acting as “sponges” or competitors. Reduced levels of miR-9, miR-125b, miR-132, miR-146a and miR-150, miR-221, and miR-222 have been reported in various HD cells, HD models as well as in postmortem HD tissues (Sinha et al., 2012; Blume et al., 2015). Therefore, it is reasonable to speculate that the interaction of lncRNAs and miRNAs may contribute to the reduced levels of these miRNAs, thereby promoting the occurrence and development of HD.

Many other lncRNAs, such as BACE1-AS,TUG1, and HAR1, ware also involved in the occurrence and development of HD although no evidence to show that they interact with miRNAs. For example, BACE1 antisense transcripts (BACE1-AS) positively regulate BACE1 mRNA levels in the brain of HD patients. It is worth of noting that knocking down of BACE1-AS,could reduce the stable mRNA level of the justice gene (Johnson, 2012). Another up-regulatedTUG1 has been reported to target p53 target (Khalil et al., 2009), a known transcriptional regulatory pathway in HD driven by the pathological activation of P53 tumor suppressor proteins. Different from BACE1-AS and TUG1, the HAR1 expression was downregulated in two brain regions cortex (Brodman region 7/9) and striatum comparing the HD autopsy to the control persons. More specifically, significant reductions in the levels of both HAR1F and HAR1R were observed in the striatum of HD patients, but not in the cortex compared with the Brodman7/9 region. Furthermore, HAR1 was considered as the target of REST, but no evidence could support this thought in a way that disruption of REST failed to downregulate HAR1 level in mice (Johnson et al., 2010).

## Conclusion

Although significant efforts have been made in dissection of pathology for the neurodegenerative disorders such as AD, PD, HD, and ALS, our understanding is still very limited. Excitingly, dramatic breakthroughs in high throughput sequencing technologies in recent years significantly speeds up our understanding on epigenetic contribution to pathogenesis of neurodegenerative disorders. Accumulated evidence has enabled us to acknowledge that lncRNAs could suppress or promote the neurodegenerative disorders *via* epigenetically regulating expression of genes crucially involved in the pathogenesis at both transcriptional and posttranscriptional levels. In the nucleus, lncRNAs can inhibit or promote gene expression through act either in *cis* or in *trans* (Wang et al., 2011). XIST is the most extensively investigated *cis-*acting lncRNA, which inactivate the whole X chromosome by inhibiting the deposition of chromatin markers and relocating this chromosome to the periphery of the nucleus (Cerase et al., 2015). LncRNA HOTAIR interacts with polycomb repressive complex2 (PRC2), leading to H3K27me3-mediated gene silencing at HOXD locus in *trans* (Rinn et al., 2007). However, the exact mechanisms of how these lncRNAs affect the onset and progression of neurodegenerative diseases remains largely unknown. On one hand, some studies on lncRNAs lack biological repeats or conducted only *in vitro*, without confirmation *in vivo*. On the other hand, although some lncRNAs are differentially expressed in in the patients and normal persons, they lack tissue specificity. For example, MEG3 has been demonstrated to be differently expressed in AD, ALS, HD and some malignant tumors. This poses a great challenge for identification of the CNS-specific lncRNAs as biomarkers. More importantly, the ceRNA theory has become the main hypothesis of how lncRNAs function as ceRNA in neurodegenerative diseases. However, it is still skeptical about whether the physiological expression level of a single lncRNA is sufficient to alter the expression level of miRNAs, because a single lncRNA represent only a very small fraction of the total miRNA targets (Denzler et al., 2014; Thomson and Dinger, 2016). Besides, since most databases were generated by using a variety of miRNA prediction algorithms, biochemical and gene expression data, hard to determine the reliability of the ceRNA interactions established from these different prediction methods (Thomson et al., 2011). In terms of the experimental strategy, the models were generated *via* ceRNA overexpression or silencing. Thus, the concern is that overexpression of miRNAs or ceRNAs at the physiological level is experimentally challenging (Liu et al., 2021). As mentioned above, most of the lncRNA studies are conducted *in vitro*, failing to mimick the *in vivo* patients. Therefore, mouse models or human brain organoids should be used as more relevant physiological model.

It is well known that a plurality of lncRNAs can target a miRNA and a single lncRNA targets multiple miRNAs simultaneously, making the interplay of miRNAs and lncRNAs extremely complicated. In this context, it is essential for the future study to investigate all the genes crucially involved in the pathogenesis as well as their regulation elements such as ncRNAs (miRNAs and lncRNAs) genetically, epigenetically, molecularly, and biochemically. Understanding the interaction networks and orchestration of all the ncRNAs and target genes could help us identify the biomarkers for diagnosis and therapy.

Antisense oligonucleotides (ASOs) have been confirmed to target lncRNAs in the nucleus more effectively than siRNAs. Furthermore, ASOs based study in disease animal models as well as the clinical trials have been conducted for some disease such as cancer. For example, specific ASO can act on specific lncRNA to alleviate the production of A β in the AD animal model (Massone et al., 2012). Moreover, Clinical trials using ASO to treat malignant tumors (Adams et al., 2017) and intrathecal injection of ASO for the therapy of ASL (Chiriboga et al., 2016) have been carried out. These studies shed light on the expectation that lncRNAs could potentially become new biomarkers for diagnosis and clinical therapy of ND, meanwhile the promising results inspire the scientists to make greater efforts to reach this goal. However, there is distance to go to identify and characterize the specific lncRNAs that contribute to the pathogenesis of neurodegenerative diseases. To this end, the animal ND models, the cell-based assays, the human brain organoids derived from ND patients and normal persons, the patients’ samples (brain tissues and blood) should be employed for analysis and validation of the specific ncRNAs. The cell-based assays and animal models such as mouse could be employed for fast and primary screening. It is worthy of noting that the large evolutionary distance between mouse and human lead to a discrepancy in genetic, anatomic, and physiological basis between animal models and humans. In addition, human brain organoids are a miniaturized and simplified version of brain organ generated *in vitro* but could recapitulate key features brain development. Thus, candidate lncRNAs identified in animal models should be further validated in human organoids even human tissues.

To sum up, further study on lncRNAs in CNSNDs will contribute to further understanding of brain function and the pathogenesis as well as the development of the promising therapeutic strategies for the CNSNDs.

## Author Contributions

SX designed the topic and conceived the structure of the manuscript. SZ, XY, YM, DS, HY, and DW collected the articles, and made the tables and figures. SZ wrote the manuscript. SX, MW, and JB revised the manuscript. All authors contributed to the manuscript.

## Conflict of Interest

The authors declare that the research was conducted in the absence of any commercial or financial relationships that could be construed as a potential conflict of interest.

## Publisher’s Note

All claims expressed in this article are solely those of the authors and do not necessarily represent those of their affiliated organizations, or those of the publisher, the editors and the reviewers. Any product that may be evaluated in this article, or claim that may be made by its manufacturer, is not guaranteed or endorsed by the publisher.

## References

[B1] Adams B. D. Parsons C. Walker L. Zhang W. C. Slack F. J. (2017). Targeting noncoding RNAs in disease. *J. Clin. Invest.* 127 761–771. 10.1172/JCI84424 28248199 PMC5330746

[B2] Balendra R. Isaacs A. M. (2018). C9orf72-mediated ALS and FTD: multiple pathways to disease. *Nat. Rev. Neurol.* 14 544–558. 10.1038/s41582-018-0047-2 30120348 PMC6417666

[B3] Bassi S. Tripathi T. Monziani A. Di Leva F. Biagioli M. (2017). Epigenetics of huntington’s disease. *Adv. Exp. Med. Biol.* 978 277–299. 10.1007/978-3-319-53889-1_15 28523552

[B4] Bastias-Candia S. Zolezzi J. M. Inestrosa N. C. (2019). Revisiting the paraquat-induced sporadic Parkinson’s disease-like model. *Mol. Neurobiol.* 56 1044–1055. 10.1007/s12035-018-1148-z 29862459

[B5] Bennett M. C. (2005). The role of alpha-synuclein in neurodegenerative diseases. *Pharmacol. Ther.* 105 311–331. 10.1016/j.pharmthera.2004.10.010 15737408

[B6] Biscarini S. Capauto D. Peruzzi G. Lu L. Colantoni A. Santini T. (2018). Characterization of the lncRNA transcriptome in mESC-derived motor neurons: Implications for FUS-ALS. *Stem Cell Res.* 27 172–179. 10.1016/j.scr.2018.01.037 29449089

[B7] Blume C. J. Hotz-Wagenblatt A. Hullein J. Sellner L. Jethwa A. Stolz T. (2015). p53-dependent non-coding RNA networks in chronic lymphocytic leukemia. *Leukemia* 29 2015–2023. 10.1038/leu.2015.119 25971364

[B8] Bose A. Beal M. F. (2016). Mitochondrial dysfunction in Parkinson’s disease. *J. Neurochem.* 139(Suppl. 1) 216–231. 10.1111/jnc.13731 27546335

[B9] Brochier C. Gaillard M. C. Diguet E. Caudy N. Dossat C. Segurens B. (2008). Quantitative gene expression profiling of mouse brain regions reveals differential transcripts conserved in human and affected in disease models. *Physiol. Genomics* 33 170–179. 10.1152/physiolgenomics.00125.2007 18252803

[B10] Calabresi P. Castrioto A. Di Filippo M. Picconi B. (2013). New experimental and clinical links between the hippocampus and the dopaminergic system in Parkinson’s disease. *Lancet Neurol.* 12 811–821. 10.1016/S1474-4422(13)70118-2 23867199

[B11] Cao B. Wang T. Qu Q. Kang T. Yang Q. (2018). Long Noncoding RNA SNHG1 promotes neuroinflammation in Parkinson’s disease via regulating miR-7/NLRP3 pathway. *Neuroscience* 388 118–127. 10.1016/j.neuroscience.2018.07.019 30031125

[B12] Causeret F. Sumia I. Pierani A. (2016). Kremen1 and Dickkopf1 control cell survival in a Wnt-independent manner. *Cell Death Differ.* 23 323–332. 10.1038/cdd.2015.100 26206087 PMC4716294

[B13] Cerase A. Pintacuda G. Tattermusch A. Avner P. (2015). Xist localization and function: new insights from multiple levels. *Genome Biol.* 16:166. 10.1186/s13059-015-0733-y 26282267 PMC4539689

[B14] Chakravarty D. Sboner A. Nair S. S. Giannopoulou E. Li R. Hennig S. (2014). The oestrogen receptor alpha-regulated lncRNA NEAT1 is a critical modulator of prostate cancer. *Nat. Commun.* 5:5383. 10.1038/ncomms6383 25415230 PMC4241506

[B15] Chalei V. Sansom S. N. Kong L. Lee S. Montiel J. F. Vance K. W. (2014). The long non-coding RNA Dali is an epigenetic regulator of neural differentiation. *Elife* 3:e04530. 10.7554/eLife.04530 25415054 PMC4383022

[B16] Chanda K. Das S. Chakraborty J. Bucha S. Maitra A. Chatterjee R. (2018). Altered levels of long NcRNAs Meg3 and neat1 in cell and animal models of huntington’s disease. *RNA Biol.* 15 1348–1363. 10.1080/15476286.2018.1534524 30321100 PMC6284602

[B17] Chen S. Liang H. Yang H. Zhou K. Xu L. Liu J. (2017). LincRNa-p21: function and mechanism in cancer. *Med. Oncol.* 34:98. 10.1007/s12032-017-0959-5 28425074

[B18] Chen Y. Lian Y. J. Ma Y. Q. Wu C. J. Zheng Y. K. Xie N. C. (2018). LncRNA SNHG1 promotes alpha-synuclein aggregation and toxicity by targeting miR-15b-5p to activate SIAH1 in human neuroblastoma SH-SY5Y cells. *Neurotoxicology* 68 212–221. 10.1016/j.neuro.2017.12.001 29217406

[B19] Chiriboga C. A. Swoboda K. J. Darras B. T. Iannaccone S. T. Montes J. De Vivo D. C. (2016). Results from a phase 1 study of nusinersen (ISIS-SMN(Rx)) in children with spinal muscular atrophy. *Neurology* 86, 890–897. 10.1212/WNL.0000000000002445 26865511 PMC4782111

[B20] Chung C. Y. Berson A. Kennerdell J. R. Sartoris A. Unger T. Porta S. (2018). Aberrant activation of non-coding RNA targets of transcriptional elongation complexes contributes to TDP-43 toxicity. *Nat. Commun.* 9:4406. 10.1038/s41467-018-06543-0 30353006 PMC6199344

[B21] Ciarlo E. Massone S. Penna I. Nizzari M. Gigoni A. Dieci G. (2013). An intronic ncRNA-dependent regulation of SORL1 expression affecting Abeta formation is upregulated in post-mortem Alzheimer’s disease brain samples. *Dis. Model Mech.* 6 424–433. 10.1242/dmm.009761 22996644 PMC3597024

[B22] Clavaguera F. Bolmont T. Crowther R. A. Abramowski D. Frank S. Probst A. (2009). Transmission and spreading of tauopathy in transgenic mouse brain. *Nat. Cell Biol.* 11 909–913. 10.1038/ncb1901 19503072 PMC2726961

[B23] Cortini F. Roma F. Villa C. (2019). Emerging roles of long non-coding RNAs in the pathogenesis of Alzheimer’s disease. *Ageing Res. Rev.* 50 19–26. 10.1016/j.arr.2019.01.001 30610928

[B24] Decressac M. Mattsson B. Weikop P. Lundblad M. Jakobsson J. Bjorklund A. (2013). TFEB-mediated autophagy rescues midbrain dopamine neurons from alpha-synuclein toxicity. *Proc. Natl. Acad. Sci. U.S.A.* 110 E1817–E1826. 10.1073/pnas.1305623110 23610405 PMC3651458

[B25] Dehay B. Bourdenx M. Gorry P. Przedborski S. Vila M. Hunot S. (2015). Targeting alpha-synuclein for treatment of Parkinson’s disease: mechanistic and therapeutic considerations. *Lancet Neurol.* 14 855–866. 10.1016/S1474-4422(15)00006-X 26050140 PMC5217462

[B26] DeJesus-Hernandez M. Finch N. A. Wang X. Gendron T. F. Bieniek K. F. Heckman M. G. (2017). In-depth clinico-pathological examination of RNA foci in a large cohort of C9ORF72 expansion carriers. *Acta Neuropathol.* 134 255–269. 10.1007/s00401-017-1725-7 28508101 PMC5508036

[B27] Denzler R. Agarwal V. Stefano J. Bartel D. P. Stoffel M. (2014). Assessing the ceRNA hypothesis with quantitative measurements of miRNA and target abundance. *Mol. Cell* 54 766–776. 10.1016/j.molcel.2014.03.045 24793693 PMC4267251

[B28] Dermentzaki G. Lotti F. (2020). New insights on the role of N (6)-Methyladenosine RNA methylation in the physiology and pathology of the nervous system. *Front. Mol. Biosci.* 7:555372. 10.3389/fmolb.2020.555372 32984403 PMC7492240

[B29] Derrien T. Johnson R. Bussotti G. Tanzer A. Djebali S. Tilgner H. (2012). The GENCODE v7 catalog of human long noncoding RNAs: analysis of their gene structure, evolution, and expression. *Genome Res.* 22 1775–1789. 10.1101/gr.132159.111 22955988 PMC3431493

[B30] Elling R. Chan J. Fitzgerald K. A. (2016). Emerging role of long noncoding RNAs as regulators of innate immune cell development and inflammatory gene expression. *Eur. J. Immunol.* 46 504–512. 10.1002/eji.201444558 26820238 PMC5404502

[B31] Faghihi M. A. Modarresi F. Khalil A. M. Wood D. E. Sahagan B. G. Morgan T. E. (2008). Expression of a noncoding RNA is elevated in Alzheimer’s disease and drives rapid feed-forward regulation of beta-secretase. *Nat. Med.* 14 723–730. 10.1038/nm1784 18587408 PMC2826895

[B32] Gagliardi S. Zucca S. Pandini C. Diamanti L. Bordoni M. Sproviero D. (2018). Long non-coding and coding RNAs characterization in peripheral blood mononuclear cells and spinal cord from amyotrophic lateral sclerosis patients. *Sci. Rep.* 8:2378. 10.1038/s41598-018-20679-5 29402919 PMC5799454

[B33] Ghavami S. Shojaei S. Yeganeh B. Ande S. R. Jangamreddy J. R. Mehrpour M. (2014). Autophagy and apoptosis dysfunction in neurodegenerative disorders. *Prog. Neurobiol.* 112 24–49. 10.1016/j.pneurobio.2013.10.004 24211851

[B34] Gu C. Chen C. Wu R. Dong T. Hu X. Yao Y. (2018). Long Noncoding RNA EBF3-AS promotes neuron apoptosis in alzheimer’s disease. *DNA Cell Biol.* 37 220–226. 10.1089/dna.2017.4012 29298096

[B35] Guo Q. Qian Z. Yan D. Li L. Huang L. (2016). LncRNA-MEG3 inhibits cell proliferation of endometrial carcinoma by repressing Notch signaling. *Biomed. Pharmacother.* 82 589–594. 10.1016/j.biopha.2016.02.049 27470401

[B36] Haeusler A. R. Donnelly C. J. Periz G. Simko E. A. Shaw P. G. Kim M. S. (2014). C9orf72 nucleotide repeat structures initiate molecular cascades of disease. *Nature* 507 195–200. 10.1038/nature13124 24598541 PMC4046618

[B37] Hegarty S. V. Green H. F. Niclis J. O’Keeffe G. W. Sullivan A. M. (2020). Editorial: the role of stem cells, epigenetics and MicroRNAs in Parkinson’s Disease. *Front. Neurosci.* 14:515. 10.3389/fnins.2020.00515 32655345 PMC7325904

[B38] Hirose T. Virnicchi G. Tanigawa A. Naganuma T. Li R. Kimura H. (2014). NEAT1 long noncoding RNA regulates transcription via protein sequestration within subnuclear bodies. *Mol. Biol. Cell* 25 169–183. 10.1091/mbc.E13-09-0558 24173718 PMC3873887

[B39] Huarte M. Guttman M. Feldser D. Garber M. Koziol M. J. Kenzelmann-Broz D. (2010). A large intergenic noncoding RNA induced by p53 mediates global gene repression in the p53 response. *Cell* 142 409–419. 10.1016/j.cell.2010.06.040 20673990 PMC2956184

[B40] Hutchinson J. N. Ensminger A. W. Clemson C. M. Lynch C. R. Lawrence J. B. Chess A. (2007). A screen for nuclear transcripts identifies two linked noncoding RNAs associated with SC35 splicing domains. *BMC Genomics* 8:39. 10.1186/1471-2164-8-39 17270048 PMC1800850

[B41] Hwang J. Y. Aromolaran K. A. Zukin R. S. (2017). The emerging field of epigenetics in neurodegeneration and neuroprotection. *Nat. Rev. Neurosci.* 18 347–361. 10.1038/nrn.2017.46 28515491 PMC6380351

[B42] Hyrskyluoto A. Bruelle C. Lundh S. H. Do H. T. Kivinen J. Rappou E. (2014). Ubiquitin-specific protease-14 reduces cellular aggregates and protects against mutant huntingtin-induced cell degeneration: involvement of the proteasome and ER stress-activated kinase IRE1alpha. *Hum. Mol. Genet.* 23 5928–5939. 10.1093/hmg/ddu317 24951540

[B43] Idogawa M. Ohashi T. Sasaki Y. Nakase H. Tokino T. (2017). Long non-coding RNA NEAT1 is a transcriptional target of p53 and modulates p53-induced transactivation and tumor-suppressor function. *Int. J. Cancer* 140 2785–2791. 10.1002/ijc.30689 28295289

[B44] Jiang J. Piao X. Hu S. Gao J. Bao M. (2020). LncRNA H19 diminishes dopaminergic neuron loss by mediating microRNA-301b-3p in Parkinson’s disease via the HPRT1-mediated Wnt/beta-catenin signaling pathway. *Aging (Albany NY)* 12 8820–8836. 10.18632/aging.102877 32434961 PMC7288916

[B45] Johnson R. (2012). Long non-coding RNAs in Huntington’s disease neurodegeneration. *Neurobiol. Dis.* 46 245–254. 10.1016/j.nbd.2011.12.006 22202438

[B46] Johnson R. Richter N. Jauch R. Gaughwin P. M. Zuccato C. Cattaneo E. (2010). Human accelerated region 1 noncoding RNA is repressed by REST in Huntington’s disease. *Physiol. Genomics* 41 269–274. 10.1152/physiolgenomics.00019.2010 20179156

[B47] Kadakkuzha B. M. Liu X. A. McCrate J. Shankar G. Rizzo V. Afinogenova A. (2015). Transcriptome analyses of adult mouse brain reveal enrichment of lncRNAs in specific brain regions and neuronal populations. *Front. Cell Neurosci.* 9:63. 10.3389/fncel.2015.00063 25798087 PMC4351618

[B48] Kaur K. Gill J. S. Bansal P. K. Deshmukh R. (2017). Neuroinflammation - A major cause for striatal dopaminergic degeneration in Parkinson’s disease. *J. Neurol. Sci.* 381 308–314. 10.1016/j.jns.2017.08.3251 28991704

[B49] Khalil A. M. Guttman M. Huarte M. Garber M. Raj A. Rivea Morales D. (2009). Many human large intergenic noncoding RNAs associate with chromatin-modifying complexes and affect gene expression. *Proc. Natl. Acad. Sci. U.S.A.* 106 11667–11672. 10.1073/pnas.0904715106 19571010 PMC2704857

[B50] Kong Q. Zhang S. Liang C. Zhang Y. Kong Q. Chen S. (2018). LncRNA XIST functions as a molecular sponge of miR-194-5p to regulate MAPK1 expression in hepatocellular carcinoma cell. *J. Cell Biochem.* 119 4458–4468. 10.1002/jcb.26540 29227532

[B51] Kovanda A. Zalar M. Sket P. Plavec J. Rogelj B. (2015). Anti-sense DNA d(GGCCCC)n expansions in C9ORF72 form i-motifs and protonated hairpins. *Sci. Rep.* 5:17944. 10.1038/srep17944 26632347 PMC4668579

[B52] Kraus T. F. J. Haider M. Spanner J. Steinmaurer M. Dietinger V. Kretzschmar H. A. (2017). Altered long noncoding RNA expression precedes the course of Parkinson’s Disease-a preliminary report. *Mol. Neurobiol.* 54 2869–2877. 10.1007/s12035-016-9854-x 27021022

[B53] Labbadia J. Morimoto R. I. (2013). Huntington’s disease: underlying molecular mechanisms and emerging concepts. *Trends Biochem. Sci.* 38 378–385. 10.1016/j.tibs.2013.05.003 23768628 PMC3955166

[B54] Lashley T. Schott J. M. Weston P. Murray C. E. Wellington H. Keshavan A. (2018). Molecular biomarkers of Alzheimer’s disease: progress and prospects. *Dis. Model Mech.* 11:dmm031781. 10.1242/dmm.031781 29739861 PMC5992610

[B55] Lee J. T. Wheeler T. C. Li L. Chin L. S. (2008). Ubiquitination of alpha-synuclein by Siah-1 promotes alpha-synuclein aggregation and apoptotic cell death. *Hum. Mol. Genet.* 17 906–917. 10.1093/hmg/ddm363 18065497

[B56] Li C. Wang X. Cai H. Fu Y. Luan Y. Wang W. (2016). Molecular microevolution and epigenetic patterns of the long non-coding gene H19 show its potential function in pig domestication and breed divergence. *BMC Evol. Biol.* 16:87. 10.1186/s12862-016-0657-5 27107967 PMC4841954

[B57] Liu S. J. Dang H. X. Lim D. A. Feng F. Y. Maher C. A. (2021). Long noncoding RNAs in cancer metastasis. *Nat. Rev. Cancer* 21 446–460. 10.1038/s41568-021-00353-1 33953369 PMC8288800

[B58] Lo Piccolo L. Yamaguchi M. (2017). RNAi of arcRNA hsromega affects sub-cellular localization of *Drosophila* FUS to drive neurodiseases. *Exp. Neurol.* 292 125–134. 10.1016/j.expneurol.2017.03.011 28342748

[B59] Luo Q. Chen Y. (2016). Long noncoding RNAs and Alzheimer’s disease. *Clin. Interv. Aging* 11 867–872. 10.2147/CIA.S107037 27418812 PMC4933566

[B60] Ma H. Lesne S. Kotilinek L. Steidl-Nichols J. V. Sherman M. Younkin L. (2007). Involvement of beta-site APP cleaving enzyme 1 (BACE1) in amyloid precursor protein-mediated enhancement of memory and activity-dependent synaptic plasticity. *Proc. Natl. Acad. Sci. U.S.A.* 104 8167–8172. 10.1073/pnas.0609521104 17470798 PMC1859992

[B61] Ma P. Li Y. Zhang W. Fang F. Sun J. Liu M. (2019). Long Non-coding RNA MALAT1 inhibits neuron apoptosis and neuroinflammation while stimulates neurite outgrowth and its correlation With MiR-125b Mediates PTGS2, CDK5 and FOXQ1 in Alzheimer’s Disease. *Curr. Alzheimer Res.* 16 596–612. 10.2174/1567205016666190725130134 31345147

[B62] Magistri M. Velmeshev D. Makhmutova M. Faghihi M. A. (2015). Transcriptomics profiling of alzheimer’s disease reveal neurovascular defects, altered amyloid-beta homeostasis, and deregulated expression of long noncoding RNAs. *J. Alzheimers Dis.* 48 647–665. 10.3233/JAD-150398 26402107 PMC4698155

[B63] Majounie E. Renton A. E. Mok K. Dopper E. G. Waite A. Rollinson S. (2012). Frequency of the C9orf72 hexanucleotide repeat expansion in patients with amyotrophic lateral sclerosis and frontotemporal dementia: a cross-sectional study. *Lancet Neurol.* 11 323–330. 10.1016/S1474-4422(12)70043-1 22406228 PMC3322422

[B64] Makhlouf M. Ouimette J. F. Oldfield A. Navarro P. Neuillet D. Rougeulle C. (2014). A prominent and conserved role for YY1 in Xist transcriptional activation. *Nat. Commun.* 5:4878. 10.1038/ncomms5878 25209548 PMC4172967

[B65] Managadze D. Lobkovsky A. E. Wolf Y. I. Shabalina S. A. Rogozin I. B. Koonin E. V. (2013). The vast, conserved mammalian lincRNome. *PLoS Comput. Biol.* 9:e1002917. 10.1371/journal.pcbi.1002917 23468607 PMC3585383

[B66] Masoumi F. Ghorbani S. Talebi F. Branton W. G. Rajaei S. Power C. (2019). Malat1 long noncoding RNA regulates inflammation and leukocyte differentiation in experimental autoimmune encephalomyelitis. *J. Neuroimmunol.* 328 50–59. 10.1016/j.jneuroim.2018.11.013 30583215

[B67] Massone S. Ciarlo E. Vella S. Nizzari M. Florio T. Russo C. (2012). NDM29, a RNA polymerase III-dependent non coding RNA, promotes amyloidogenic processing of APP and amyloid beta secretion. *Biochim. Biophys. Acta* 1823, 1170–1177. 10.1016/j.bbamcr.2012.05.001 22580042

[B68] Mateos-Aparicio P. Rodriguez-Moreno A. (2019). The impact of studying brain plasticity. *Front. Cell Neurosci.* 13:66. 10.3389/fncel.2019.00066 30873009 PMC6400842

[B69] Militello G. Hosen M. R. Ponomareva Y. Gellert P. Weirick T. John D. (2018). A novel long non-coding RNA Myolinc regulates myogenesis through TDP-43 and Filip1. *J. Mol. Cell Biol.* 10 102–117. 10.1093/jmcb/mjy025 29618024 PMC7191624

[B70] Modarresi F. Faghihi M. A. Lopez-Toledano M. A. Fatemi R. P. Magistri M. Brothers S. P. (2012). Inhibition of natural antisense transcripts in vivo results in gene-specific transcriptional upregulation. *Nat. Biotechnol.* 30 453–459. 10.1038/nbt.2158 22446693 PMC4144683

[B71] Modarresi F. Faghihi M. A. Patel N. S. Sahagan B. G. Wahlestedt C. Lopez-Toledano M. A. (2011). Knockdown of BACE1-AS nonprotein-coding transcript modulates beta-amyloid-related hippocampal neurogenesis. *Int. J. Alzheimers Dis.* 2011:929042. 10.4061/2011/929042 21785702 PMC3139208

[B72] More S. Choi D. K. (2017). Neuroprotective role of Atractylenolide-I in an *In Vitro* and *In Vivo* model of Parkinson’s disease. *Nutrients* 9:451. 10.3390/nu9050451 28468332 PMC5452181

[B73] Mori K. Weng S. M. Arzberger T. May S. Rentzsch K. Kremmer E. (2013). The C9orf72 GGGGCC repeat is translated into aggregating dipeptide-repeat proteins in FTLD/ALS. *Science* 339 1335–1338. 10.1126/science.1232927 23393093

[B74] Naganuma T. Nakagawa S. Tanigawa A. Sasaki Y. F. Goshima N. Hirose T. (2012). Alternative 3’-end processing of long noncoding RNA initiates construction of nuclear paraspeckles. *EMBO J.* 31 4020–4034. 10.1038/emboj.2012.251 22960638 PMC3474925

[B75] Peng T. Liu X. Wang J. Liu Y. Fu Z. Ma X. (2019). Long noncoding RNA HAGLROS regulates apoptosis and autophagy in Parkinson’s disease via regulating miR-100/ATG10 axis and PI3K/Akt/mTOR pathway activation. *Artif. Cells Nanomed. Biotechnol.* 47 2764–2774. 10.1080/21691401.2019.1636805 31298038

[B76] Pizzorusso T. Tognini P. (2020). Interplay between metabolism, nutrition and epigenetics in shaping brain DNA methylation, neural function and behavior. *Genes (Basel)* 11:742. 10.3390/genes11070742 32635190 PMC7397264

[B77] Pollard K. S. Salama S. R. Lambert N. Lambot M. A. Coppens S. Pedersen J. S. (2006). An RNA gene expressed during cortical development evolved rapidly in humans. *Nature* 443 167–172. 10.1038/nature05113 16915236

[B78] Ponjavic J. Oliver P. L. Lunter G. Ponting C. P. (2009). Genomic and transcriptional co-localization of protein-coding and long non-coding RNA pairs in the developing brain. *PLoS Genet.* 5:e1000617. 10.1371/journal.pgen.1000617 19696892 PMC2722021

[B79] Qian C. Ye Y. Mao H. Yao L. Sun X. Wang B. (2019). Downregulated lncRNA-SNHG1 enhances autophagy and prevents cell death through the miR-221/222/p27/mTOR pathway in Parkinson’s disease. *Exp. Cell Res.* 384 111614. 10.1016/j.yexcr.2019.111614 31499060

[B80] Quan Z. Zheng D. Qing H. (2017). Regulatory roles of long non-coding RNAs in the central nervous system and associated neurodegenerative diseases. *Front. Cell. Neurosci.* 11:175. 10.3389/fncel.2017.00175 28713244 PMC5491930

[B81] Quinn J. J. Chang H. Y. (2015). Unique features of long non-coding RNA biogenesis and function. *Nat. Rev. Genet.* 17 47–62. 10.1038/nrg.2015.10 26666209

[B82] Reddy K. Zamiri B. Stanley S. Y. Macgregor R. B. Jr. Pearson C. E. (2013). The disease-associated r(GGGGCC)n repeat from the C9orf72 gene forms tract length-dependent uni- and multimolecular RNA G-quadruplex structures. *J. Biol. Chem.* 288 9860–9866. 10.1074/jbc.C113.452532 23423380 PMC3617286

[B83] Renton A. E. Chio A. Traynor B. J. (2014). State of play in amyotrophic lateral sclerosis genetics. *Nat. Neurosci.* 17 17–23. 10.1038/nn.3584 24369373 PMC4544832

[B84] Rinn J. L. Kertesz M. Wang J. K. Squazzo S. L. Xu X. Brugmann S. A. (2007). Functional demarcation of active and silent chromatin domains in human HOX loci by noncoding RNAs. *Cell* 129 1311–1323. 10.1016/j.cell.2007.05.022 17604720 PMC2084369

[B85] Ross C. A. Tabrizi S. J. (2011). Huntington’s disease: from molecular pathogenesis to clinical treatment. *Lancet Neurol.* 10 83–98. 10.1016/S1474-4422(10)70245-3 21163446

[B86] Ryan P. Patel B. Makwana V. Jadhav H. R. Kiefel M. Davey A. (2018). Peptides, peptidomimetics, and carbohydrate-peptide conjugates as amyloidogenic aggregation inhibitors for alzheimer’s disease. *ACS Chem. Neurosci.* 9 1530–1551. 10.1021/acschemneuro.8b00185 29782794

[B87] Salmena L. Poliseno L. Tay Y. Kats L. Pandolfi P. P. (2011). A ceRNA hypothesis: the Rosetta Stone of a hidden RNA language? *Cell* 146 353–358. 10.1016/j.cell.2011.07.014 21802130 PMC3235919

[B88] Sareen D. O’Rourke J. G. Meera P. Muhammad A. K. Grant S. Simpkinson M. (2013). Targeting RNA foci in iPSC-derived motor neurons from ALS patients with a C9ORF72 repeat expansion. *Sci. Transl. Med.* 5:208ra149. 10.1126/scitranslmed.3007529 24154603 PMC4090945

[B89] Shah K. Desilva S. Abbruscato T. (2012). The role of glucose transporters in brain disease: diabetes and Alzheimer’s Disease. *Int. J. Mol. Sci.* 13 12629–12655. 10.3390/ijms131012629 23202918 PMC3497292

[B90] Shelkovnikova T. A. Kukharsky M. S. An H. Dimasi P. Alexeeva S. Shabir O. (2018). Protective paraspeckle hyper-assembly downstream of TDP-43 loss of function in amyotrophic lateral sclerosis. *Mol. Neurodegener.* 13:30. 10.1186/s13024-018-0263-7 29859124 PMC5984788

[B91] Simchovitz A. Hanan M. Niederhoffer N. Madrer N. Yayon N. Bennett E. R. (2019). NEAT1 is overexpressed in Parkinson’s disease substantia nigra and confers drug-inducible neuroprotection from oxidative stress. *FASEB J.* 33 11223–11234. 10.1096/fj.201900830R 31311324 PMC6766647

[B92] Sinha M. Mukhopadhyay S. Bhattacharyya N. P. (2012). Mechanism(s) of alteration of micro RNA expressions in Huntington’s disease and their possible contributions to the observed cellular and molecular dysfunctions in the disease. *Neuromolecular. Med.* 14 221–243. 10.1007/s12017-012-8183-0 22581158

[B93] Song Q. Geng Y. Li Y. Wang L. Qin J. (2019). Long noncoding RNA NORAD regulates MPP+-induced Parkinson’s disease model cells. *J. Chem. Neuroanat.* 101:101668. 10.1016/j.jchemneu.2019.101668 31421205

[B94] Sosinska P. Mikula-Pietrasik J. Ksiazek K. (2015). The double-edged sword of long non-coding RNA: the role of human brain-specific BC200 RNA in translational control, neurodegenerative diseases, and cancer. *Mutat. Res. Rev. Mutat. Res.* 766 58–67. 10.1016/j.mrrev.2015.08.002 26596549

[B95] Sun J. Pan L. M. Chen L. B. Wang Y. (2017). LncRNA XIST promotes human lung adenocarcinoma cells to cisplatin resistance via let-7i/BAG-1 axis. *Cell Cycle* 16 2100–2107. 10.1080/15384101.2017.1361071 28961027 PMC5731406

[B96] Sunwoo J. S. Lee S. T. Im W. Lee M. Byun J. I. Jung K. H. (2017). Altered expression of the long noncoding RNA NEAT1 in huntington’s disease. *Mol. Neurobiol.* 54 1577–1586. 10.1007/s12035-016-9928-9 27221610

[B97] Sutherland G. Mellick G. Newman J. Double K. L. Stevens J. Lee L. (2008). Haplotype analysis of the IGF2-INS-TH gene cluster in Parkinson’s disease. *Am. J. Med. Genet. B Neuropsychiatr. Genet.* 147B 495–499. 10.1002/ajmg.b.30633 18085551

[B98] Tang Y. Le W. (2016). Differential roles of M1 and M2 microglia in neurodegenerative diseases. *Mol. Neurobiol.* 53 1181–1194. 10.1007/s12035-014-9070-5 25598354

[B99] Tatton W. G. Chalmers-Redman R. Brown D. Tatton N. (2003). Apoptosis in Parkinson’s disease: signals for neuronal degradation. *Ann. Neurol.* 53 Suppl 3 S61–S70. 10.1002/ana.10489 12666099

[B100] Thompson C. Otero P. Srinageshwar B. Petersen R. B. Dunbar G. L. Rossignol J. (2020). Possible roles of epigenetics in stem cell therapy for Parkinson’s disease. *Epigenomics* 12 647–656. 10.2217/epi-2019-0347 32396465

[B101] Thomson D. W. Bracken C. P. Goodall G. J. (2011). Experimental strategies for microRNA target identification. *Nucleic Acids Res.* 39 6845–6853. 10.1093/nar/gkr330 21652644 PMC3167600

[B102] Thomson D. W. Dinger M. E. (2016). Endogenous microRNA sponges: evidence and controversy. *Nat. Rev. Genet.* 17 272–283. 10.1038/nrg.2016.20 27040487

[B103] Tollervey J. R. Curk T. Rogelj B. Briese M. Cereda M. Kayikci M. (2011). Characterizing the RNA targets and position-dependent splicing regulation by TDP-43. *Nat. Neurosci.* 14 452–458. 10.1038/nn.2778 21358640 PMC3108889

[B104] Ulitsky I. Bartel D. P. (2013). lincRNAs: genomics, evolution, and mechanisms. *Cell* 154 26–46. 10.1016/j.cell.2013.06.020 23827673 PMC3924787

[B105] Vangoor V. R. Gomes-Duarte A. Pasterkamp R. J. (2020). Long non-coding RNAs in motor neuron development and disease. *J. Neurochem.* 156, 777–801. 10.1111/jnc.15198 32970857 PMC8048821

[B106] Wang H. Lu B. Chen J. (2019). Knockdown of lncRNA SNHG1 attenuated Abeta25-35-inudced neuronal injury via regulating KREMEN1 by acting as a ceRNA of miR-137 in neuronal cells. *Biochem. Biophys. Res. Commun.* 518 438–444. 10.1016/j.bbrc.2019.08.033 31447119

[B107] Wang K. C. Yang Y. W. Liu B. Sanyal A. Corces-Zimmerman R. Chen Y. (2011). A long noncoding RNA maintains active chromatin to coordinate homeotic gene expression. *Nature* 472 120–124. 10.1038/nature09819 21423168 PMC3670758

[B108] West J. A. Davis C. P. Sunwoo H. Simon M. D. Sadreyev R. I. Wang P. I. (2014). The long noncoding RNAs NEAT1 and MALAT1 bind active chromatin sites. *Mol. Cell* 55 791–802. 10.1016/j.molcel.2014.07.012 25155612 PMC4428586

[B109] Wu Y. Y. Kuo H. C. (2020). Functional roles and networks of non-coding RNAs in the pathogenesis of neurodegenerative diseases. *J. Biomed. Sci.* 27:49. 10.1186/s12929-020-00636-z 32264890 PMC7140545

[B110] Xu W. Li K. Fan Q. Zong B. Han L. (2020a). Knockdown of long non-coding RNA SOX21-AS1 attenuates amyloid-beta-induced neuronal damage by sponging miR-107. *Biosci. Rep.* 40:BSR20194295. 10.1042/BSR20194295 32124921 PMC7103586

[B111] Xu W. Zhang L. Geng Y. Liu Y. Zhang N. (2020b). Long noncoding RNA GAS5 promotes microglial inflammatory response in Parkinson’s disease by regulating NLRP3 pathway through sponging miR-223-3p. *Int. Immunopharmacol.* 85:106614. 10.1016/j.intimp.2020.106614 32470877

[B112] Xu X. Zhuang C. Wu Z. Qiu H. Feng H. Wu J. (2018). LincRNA-p21 inhibits cell viability and promotes cell apoptosis in Parkinson’s Disease through Activating alpha-Synuclein expression. *Biomed. Res. Int.* 2018:8181374. 10.1155/2018/8181374 30671473 PMC6323514

[B113] Yan Y. Yan H. Teng Y. Wang Q. Yang P. Zhang L. (2020). Long non-coding RNA 00507/miRNA-181c-5p/TTBK1/MAPT axis regulates tau hyperphosphorylation in Alzheimer’s disease. *J. Gene Med.* 22:e3268. 10.1002/jgm.3268 32891070

[B114] Yang J. H. Li J. H. Jiang S. Zhou H. Qu L. H. (2013). ChIPBase: a database for decoding the transcriptional regulation of long non-coding RNA and microRNA genes from ChIP-Seq data. *Nucleic Acids Res.* 41 D177–D187. 10.1093/nar/gks1060 23161675 PMC3531181

[B115] Yang S. Yang H. Luo Y. Deng X. Zhou Y. Hu B. (2021). Long non-coding RNAs in neurodegenerative diseases. *Neurochem. Int.* 148:105096. 10.1016/j.neuint.2021.105096 34118305

[B116] Yen Y. P. Hsieh W. F. Tsai Y. Y. Lu Y. L. Liau E. S. Hsu H. C. (2018). Dlk1-Dio3 locus-derived lncRNAs perpetuate postmitotic motor neuron cell fate and subtype identity. *Elife* 7:38080. 10.7554/eLife.38080 30311912 PMC6221546

[B117] Yi J. Chen B. Yao X. Lei Y. Ou F. Huang F. (2019). Upregulation of the lncRNA MEG3 improves cognitive impairment, alleviates neuronal damage, and inhibits activation of astrocytes in hippocampus tissues in Alzheimer’s disease through inactivating the PI3K/Akt signaling pathway. *J. Cell Biochem.* 120 18053–18065. 10.1002/jcb.29108 31190362

[B118] Yue D. Guanqun G. Jingxin L. Sen S. Shuang L. Yan S. (2019). Silencing of long noncoding RNA XIST attenuated Alzheimer’s disease-related BACE1 alteration through miR-124. *Cell Biol. Int.* 44 630–636. 10.1002/cbin.11263 31743528

[B119] Zempel H. Mandelkow E. M. (2015). Tau missorting and spastin-induced microtubule disruption in neurodegeneration: alzheimer disease and hereditary spastic paraplegia. *Mol. Neurodegener.* 10:68. 10.1186/s13024-015-0064-1 26691836 PMC4687341

[B120] Zhang J. Yao T. Wang Y. Yu J. Liu Y. Lin Z. (2016a). Long noncoding RNA MEG3 is downregulated in cervical cancer and affects cell proliferation and apoptosis by regulating miR-21. *Cancer Biol. Ther.* 17 104–113. 10.1080/15384047.2015.1108496 26574780 PMC4847830

[B121] Zhang K. Donnelly C. J. Haeusler A. R. Grima J. C. Machamer J. B. Steinwald P. (2015). The C9orf72 repeat expansion disrupts nucleocytoplasmic transport. *Nature* 525 56–61. 10.1038/nature14973 26308891 PMC4800742

[B122] Zhang L. Fang Y. Cheng X. Lian Y. J. Xu H. L. (2019a). Silencing of long noncoding RNA SOX21-AS1 relieves neuronal oxidative stress injury in mice with alzheimer’s disease by upregulating FZD3/5 via the Wnt signaling pathway. *Mol. Neurobiol.* 56 3522–3537. 10.1007/s12035-018-1299-y 30143969

[B123] Zhang L. M. Wang M. H. Yang H. C. Tian T. Sun G. F. Ji Y. F. (2019b). Dopaminergic neuron injury in Parkinson’s disease is mitigated by interfering lncRNA SNHG14 expression to regulate the miR-133b/alpha-synuclein pathway. *Aging (Albany NY)* 11 9264–9279. 10.18632/aging.102330 31683259 PMC6874444

[B124] Zhang Q. S. Wang Z. H. Zhang J. L. Duan Y. L. Li G. F. Zheng D. L. (2016b). Beta-asarone protects against MPTP-induced Parkinson’s disease via regulating long non-coding RNA MALAT1 and inhibiting alpha-synuclein protein expression. *Biomed. Pharmacother.* 83 153–159. 10.1016/j.biopha.2016.06.017 27470562

[B125] Zhao Y. Wang Z. Mao Y. Li B. Zhu Y. Zhang S. (2020). NEAT1 regulates microtubule stabilization via FZD3/GSK3beta/P-tau pathway in SH-SY5Y cells and APP/PS1 mice. *Aging (Albany NY)* 12 23233–23250. 10.18632/aging.104098 33221742 PMC7746375

[B126] Zhou T. Qin G. Yang L. Xiang D. Li S. (2019). LncRNA XIST regulates myocardial infarction by targeting miR-130a-3p. *J. Cell Physiol.* 234 8659–8667. 10.1002/jcp.26327 29226319

[B127] Zhu J. Zhang R. Yang D. Li J. Yan X. Jin K. (2018). Knockdown of long non-coding RNA XIST inhibited doxorubicin resistance in colorectal cancer by upregulation of miR-124 and Downregulation of SGK1. *Cell Physiol. Biochem.* 51 113–128. 10.1159/000495168 30439718

[B128] Zimmer-Bensch G. (2019). Emerging roles of long non-coding RNAs as drivers of brain evolution. *Cells* 8:1399. 10.3390/cells8111399 31698782 PMC6912723

